# Protein kinase C delta null mice exhibit structural alterations in articular surface, intra-articular and subchondral compartments

**DOI:** 10.1186/s13075-015-0720-4

**Published:** 2015-08-17

**Authors:** Xiaohong Yang, Dian Teguh, Jian-Ping Wu, Bo He, Thomas Brett Kirk, Shengnan Qin, Siming Li, Honghui Chen, Wei Xue, Benjamin Ng, Shek Man Chim, Jennifer Tickner, Jiake Xu

**Affiliations:** Guangzhou Institute of Traumatic Surgery, the Fourth Affiliated Hospital of Medical College, Jinan University, Guangzhou, 510220 China; School of Pathology and Laboratory Medicine, University of Western Australia, Perth, WA 6009 Australia; Department of Mechanical Engineering, Curtin University, Perth, WA 6102 Australia; Department of Biomedical Engineering, Key Laboratory of Biomaterials of Guangdong Higher Education Institutes, Jinan University, Guangzhou, 510632 China; Present address: Harry Perkins Institute of Medical Research, The University of Western Australia, Nedlands, Perth, WA 6009 Australia

## Abstract

**Introduction:**

Structural alterations in intra-articular and subchondral compartments are hallmarks of osteoarthritis, a degenerative disease that causes pain and disability in the aging population. Protein kinase C delta (PKC-δ) plays versatile functions in cell growth and differentiation, but its role in the articular cartilage and subchondral bone is not known.

**Methods:**

Histological analysis including alcian blue, safranin O staining and fluorochrome labeling were used to reveal structural alterations at the articular cartilage surface and bone–cartilage interface in PKC-δ knockout (KO) mice. The morphology and organization of chondrocytes were studied using confocal microscopy. Glycosaminoglycan content was studied by micromass culture of chondrocytes of PKC-δ KO mice.

**Results:**

We uncovered atypical structural demarcation between articular cartilage and subchondral bone of PKC-δ KO mice. Histology analyses revealed a thickening of the articular cartilage and calcified bone–cartilage interface, and decreased safranin O staining accompanied by an increase in the number of hypertrophic chondrocytes in the articular cartilage of PKC-δ KO mice. Interestingly, loss of demarcation between articular cartilage and bone was concomitant with irregular chondrocyte morphology and arrangement. Consistently, in vivo calcein labeling assay showed an increased intensity of calcein labeling in the interface of the growth plate and metaphysis in PKC-δ KO mice. Furthermore, in vitro culture of chondrocyte micromass showed a decreased alcian blue staining of chondrocyte micromass in the PKC-δ KO mice, indicative of a reduced level of glycosaminoglycan production.

**Conclusions:**

Our data imply a role for PKC-δ in the osteochondral plasticity of the interface between articular cartilage and the osteochondral junction.

**Electronic supplementary material:**

The online version of this article (doi:10.1186/s13075-015-0720-4) contains supplementary material, which is available to authorized users.

## Introduction

Osteoarthritis (OA) is the most prevalent degenerative joint disease in the aging population with clinical joint dysfunction characterized by pain, joint instability and loss of motion [1]. The underlining pathogenesis of OA is not well understood, but both genetic and environmental factors are involved [2–4]. Although OA has been traditionally classified as a disease of cartilage, it affects the whole joint as a functional unit that encompasses the articular cartilage and the subchondral bone. The development of OA is clearly associated with early atypical structure modification of the articular cartilage and the calcified bone–cartilage interface in which cartilage and bone merge over the calcified tissue barrier. In particular, alterations of osteochondral plasticity occur early during the development of OA and thus have been attracting interest as potentially underlying the pathogenesis of OA [5].

Protein kinase C delta (PKC-δ) is a Ser/Thr kinase which is ubiquitously expressed in mammalian cells and classified as a novel member of the PKC subgroup that comprises PKC-δ, −ε, −η, and -θ. Multiple lines of evidence indicate that PKC-δ is activated in distinct ways that regulate cellular functions such as the control of growth, differentiation and apoptosis [6]. Genetic knockout (KO) studies have found that PKC-δ is involved in regulating B cell proliferation [7] and vein graft arteriosclerosis [8]. PKC-δ has also been reported to play a part in bone and cartilage biology [9]. It regulates osteoclastic bone resorption [10–12] and embryonic bone formation [13]. PKC-δ KO mice displayed an Osteopetrotic phenotype evidenced by an increase in the bone volume [10, 11], and were resistant to disc degeneration [14]. However, its role in articular cartilage and the bone–cartilage interface in adult mice is still not known.

In this study, using PKC-δ KO mice, we examined the role of PKC-δ in the structural integrity of articular cartilage, and the bone–cartilage interface. The results showed atypical structural alterations in the articular cartilage and subchondral bone, with abnormal chondrocyte morphology in PKC-δ KO mice, featuring a composition shift in the balance of articular cartilage to calcified subchondral bone. Understanding the role of PKC-δ in the pathological changes at the osteochondral junction may help to explore potential therapeutic applications for OA.

## Methods

### Generation of PKC-δ KO mice

PKC-δ KO mice were originally generated by Miyamoto et al. [7]. The mice were backcrossed to a C57BL/6 background [11]. Littermates of PKC-δ KO mice and wild-type (WT) mice at 12 weeks were used in this study. All animal handling procedures complied with National Health and Medical Research Council Guidelines and were approved by the Animal Ethics Committee (AEC No. 3/100/755) of the University of Western Australia, Perth, Western Australia, Australia.

### Histology and staining

Tibia (with tibial condyle) and femur (with femoral condyle) from five WT and five PKC-δ KO mice were dissected and fixed in 10 % neutral buffered formalin (NBF) for 24 hours. The samples were decalcified in 9 % formic acid for 3 to 5 days, embedded in paraffin and cut into 5-μm thick sections. Hard tissue specimens were prepared using standard procedures which include dehydration, clearing and methylmethacrylate (MMA) embedding as previously described [15]. Alcian blue staining, safranin O staining, Ponceau 2R staining and direct red polarized light staining were performed as described previously [11]. Images were collected using Zeiss and Axio Vision 4.5 microscopic image analysis system (Zeiss, Oberkochen, Germany). For height measurement of the alcian blue staining in growth plate regions, three measurements were taken: one in the middle of the growth plate, and one at either end of the growth plate for each mouse (n = 5).

### Fluorochrome labeling analysis

Fluorochrome labeling was performed using sterilized calcein (MP Biomedical, Sydney, Australia) and was administered to five KO and five WT mice by intraperitoneal injection at a dose of 5 mg/kg. A second calcein injection was performed 5 days after the first injection. Mice were sacrificed 2 days after the second injection. The hindlimbs from age- and sex-matched WT and PKC-δ KO mice were fixed in ethanol, MMA embedded and sectioned as previously described [15]. Fluorescence was visualized by confocal microscopy, and fluorescently labeled intensity versus areas (μm^2^) in trabecular bone were measured and calculated using LSM 510 Release Version 4.0 SP 2 (excitation 488 nm, emission 520 nm; Zeiss) [15].

### Fiber optic laser scanning confocal microscopy

Fiber optic laser scanning confocal microscopy (FOLSCM; Optiscan Pty Ltd, Melbourne, Australia) uses an optic fiber to deliver laser to a region of interest (ROI) of a specimen stained fluorescently [16]. The fluorescent light emissions from the ROI were collected and transmitted by the optic fiber to the photodetector to generate a digital image. The imaging mechanism of FOLSCM used in this study has allowed the development of confocal arthroscopy for studying the internal microstructure of articular cartilage without biopsy [17]. In this study, each mouse femoral condyle was stained with 0.03 g/L acriflavine solution for 3 minutes. After washing thoroughly with phosphate-buffered saline (PBS), the femoral condyle was placed into a specially designed apparatus to keep the tissue hydrated while acquiring a confocal image series of the articular cartilage in the central weight-bearing regions using FOLSCM. The acquisition of confocal microscopic images was conducted using a 488 nm excitation laser and 40×/1.2 Olympus PlanApo objective lens (Optiscan Pty Ltd, Melbourne, Australia); the image step was set at 1 μm. After confocal image stacks were acquired, they were reconstructed into a three-dimensional image using VoxBlast software (VayTek Inc., Fairfield, Iowa, USA), and a maximum brightness image (MBI) generated using the computer software F900e, proprietary to the confocal microscope. A MBI contains the maximum pixel value for each *xy* location from all the two-dimesional image slices [16] and represents a view of all of the data in the confocal image stacks combined into a single image showing only in-focus image information.

### Isolation and culture of mouse primary articular chondrocytes

Three-week-old WT and KO mice were sacrificed and the femoral heads, femoral condyles and tibial plateau were collected in complete culture medium consisting of Dulbecco’s modified Eagle’s medium (DMEM) supplemented with 1× GlutaMAX™, 20 U/mL penicillin, 20 μg/mL streptomycin and 10 % (v/v) fetal bovine serum (Gibco, Life Technologies, Grand Island, NY, USA). The tissues were transferred into a sterile 100-mm petri dish containing 12 mL collagenase type IV (Gibco, Life Technologies) (3 mg/mL) in cell culture medium (consisting only of DMEM supplemented with 1× GlutaMAX™, 20 U/mL penicillin and 20 μg/mL streptomycin) and incubated for 45 minutes at 37 °C. The tissue fragments were agitated for 30 seconds before the pieces were transferred to a new sterile 100-mm petri dish containing fresh collagenase type IV solution. The incubation and agitation steps were repeated before the tissue fragments were transferred into a new sterile 100-mm petri dish containing 20 mL collagenase type IV (0.5 mg/mL) to incubate overnight at 37 °C. The following day, the tissue fragments were vigorously agitated and allowed to sit for 2 minutes at room temperature. The resultant cell suspension was strained through a 100-μm cell strainer and centrifuged for 10 minutes at 200 × g. The cell pellet was resuspended in 1 mL complete culture medium and directly seeded at the density of 2 × 10^5^ cells in a micromass (20 μL) on a 48-well plate. The cells were incubated for 2 hours at 37 °C at 5 % CO_2_. After 2 hours, complete culture medium supplemented with 50 μg/mL ascorbic acid and 10 nM dexamethasone was added gently into the well and the cells were returned to the incubator. Culture media was replaced every 2 days for 6 days. The micromass was rinsed gently with sterile PBS pH 7.4 and fixed with 2.5 % glutaraldehyde for 10 minutes. The fixed micromass was stained with alcian blue solution (1 % w/v in 3 % glacial acetic solution, pH 1.00) for 30 minutes and washed with tap water until the excess stain was rinsed off. Nuclei number was determined by counterstaining with DAPI nuclear stain and visualizing using a fluorescence inverted microscope (Nikon Ti, Coherent Scientific, Adelaide, Australia).

### Statistical analysis

Single comparison tests were performed using a two-tailed Students *t*-test using STATA software (Statacorp, College Station, Texas, USA). For comparisons between multiple means, a one-way analysis of variance (Bonferroni post-hoc test) was used. *p*-values <0.05 were considered significant. All charts and data are represented as mean ± standard deviation (SD).

## Results

### PKC-δ KO mice display atypical structural alterations at the articular cartilage and bone–cartilage interface

**Fig. 1:**
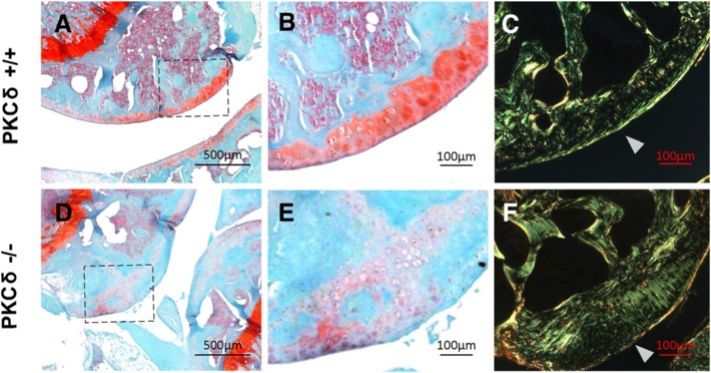
Histological examination of safranin O staining and polarized light microscopy analysis of cartilage from protein kinase C delta (PKC-δ)+/+ and PKC-δ−/− mice. **a**, **d** Representative images of articular cartilage stained with safranin O (50×). **b**, **e** Higher magnification images of dashed square area of images **a** and **d** respectively (200×). **c**, **f** Polarized images of direct red stained articular cartilage of the approximate location of **b** and **e**, respectively

Using histology with safranin O staining, we first observed a decreased safranin O staining in the articular cartilage surface in PKC-δ KO mice (Fig. 1d,e) as compared to WT mice (Fig. 1a,b). Interestingly, we also observed a thickening of articular cartilage and calcified bone–cartilage interface in the PKC-δ KO mice (Fig. 1d,e) as compared to those from WT mice (Fig. 1a,b). The thickening of articular cartilage and calcified bone–cartilage interface was further confirmed using polarized imaging in the PKC-δ KO mice (Fig. 1f) as compared to WT mice (Fig. 1c). In addition, the polarized image stained with direct red revealed an irregular articular cartilage surface in PKC-δ KO mice, with all layers of chondrocytes exhibiting disorganization, uneven size and lack of normal hierarchy (Fig. 1f), as compared to the articular cartilage surface in WT mice (Fig. 1c).

### PKC-δ KO mice exhibit disorganized chondrocyte morphology

**Fig. 2:**
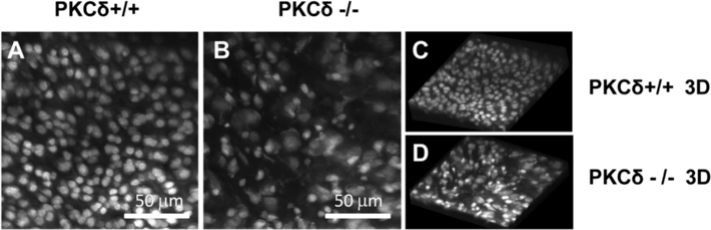
Confocal microscopic examination of chondrocytes indicated there is a significant morphological difference between the chondrocytes of protein kinase C delta (PKC-δ)+/+ (**a**, **c**) and PKC-δ−/− (**b**, **d**). **a**, **b** Chondrocyte morphology revealed by a MBI reconstructed from the confocal image stacks of PKC-δ+/+ and PKC-δ−/− mice, respectively. **c**, **d** Three-dimensional (3D) images reconstructed from the confocal image stacks using Voxblast. (Field of view: 150 μm × 150 μm)

Histological analysis further revealed an increased number of hypertrophic chondrocytes with irregular chondrocyte arrangement in PKC-δ KO mice (Fig. 1b,d). Strikingly, using FOLSCM analysis, we further observed alterations in cell morphology and arrangement of chondrocytes in PKC-δ KO mice (Fig. 2b,d) in comparison with the WT mice (Fig. 2a,c). The chondrocytes in the PKC-δ KO mice display irregular shapes as compared to those in WT articular cartilage. In addition, there is also an apparent reduction in the numbers of chondrocytes in the PKC-δ KO mice.

### Increased in vivo calcein labeling intensity in the interface between growth plate and metaphysis in PKC-δ KO mice

**Fig. 3:**
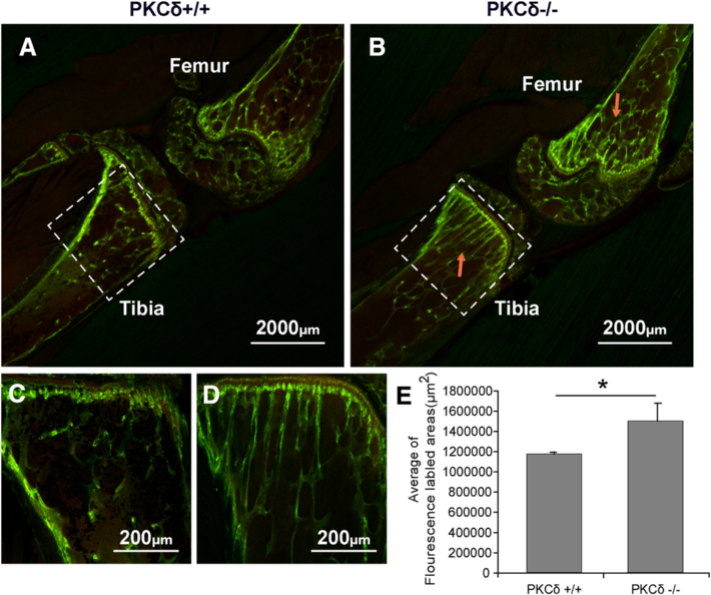
Confocal microscopic analyses of calcein fluorescence revealed an enhanced bone formation in the interface between the growth plate and metaphysis in protein kinase C delta (PKC-δ)−/− mice. **a**, **b** The tibia and femur trabecular bone showed an increased fluorescence (indicated by red arrow) in the PKC-δ−/− mice (**b**) as compared to PKC-δ+/+ mice (**a**). **c**, **d** An enlargement of the tibia from panels **a** and **b** respectively. **e** The measurement of fluorescently labeled intensity per area (μm^2^) of trabecular bone from panels **c** and **d**. *p < 0.05. (Laser scanning confocal microscope: **a** and **b**, ×12.5; **c** and **d**, ×100)

**Fig. 4:**
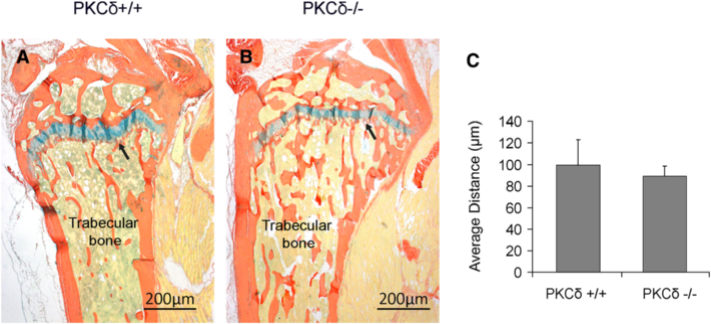
Histological analysis of the interface between the growth plate and metaphysis in protein kinase C delta (PKC-δ)+/+ and PKC-δ−/− mice. **a**, **b** Alcian blue staining was performed in tibia with Ponceau S counterstain. Arrow points to the area where the matrix of the growth plate is located. **c** Measurement of the height of growth plates by alcian blue staining regions in PKC-δ+/+ and PKC-δ−/− mice

Next, we evaluated in vivo calcein labeling-based bone formation in the interface between the growth plate and metaphysis by measuring calcein labeling intensity as described in the Materials and methods [15]. The results showed that there is a significant increase in calcein labeling intensity per area (μm^2^) in PKC-δ KO mice as compared to WT mice, suggesting an increase in bone formation in the interface between the growth plate and metaphysis in PKC-δ KO mice (Fig. 3). Histological examination revealed that there is slightly weaker staining with alcian blue (Fig. 4) and safranin O (Additional file 1) in PKC-δ KO mice, with no significant difference in the height of the growth plate as measured by alcian blue (Fig. 4) and safranin O staining regions (Additional file 1). Ponceau 2R staining showed increased trabecular bone (Fig. 4), which is consistent with our previous observations using hematoxylin and eosin staining [11].

### Micromass cultures of PKC-δ KO chondrocytes have decreased glycosaminoglycan content

**Fig. 5:**
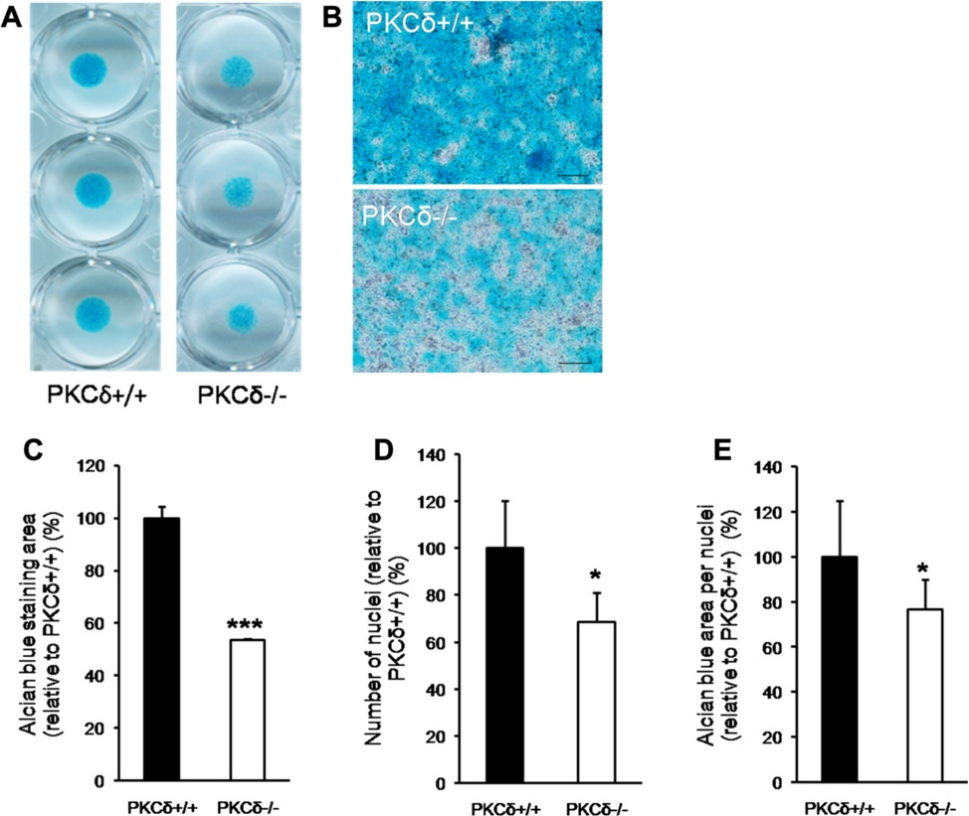
Chondrocytes from protein kinase C delta (PKC-δ)−/− mice have defective proliferation and matrix production *in vitro* compared to chondrocytes from PKC-δ+/+ mice. **a** Low magnification scanned images of alcian blue-stained PKC-δ+/+ and PKC-δ−/− chondrocyte micromass cultures. **b** 100× magnified images showing the difference in the area of alcian blue staining in PKC-δ+/+ versus PKC-δ−/− chondrocyte micromass cultures (scale bar 100μm). **c** Analysis of alcian blue staining area in PKC-δ+/+ and PKC-δ−/− micromass cultures relative to PKC-δ+/+ (n = 5, ***p < 0.001). **d** Analysis of cell numbers in PKC-δ+/+ and PKCδ−/− micromass cultures relative to PKC-δ+/+ (n = 5, *p < 0.05). **e** Analysis of alcian blue staining area per cell in PKC-δ+/+ and PKC-δ−/− relative to in PKC-δ+/+ (n = 5, *p < 0.05)

Finally, we examined synthesis ability of chondrocytes of PKC-δ KO mice using micromass culture and alcian blue staining. The results showed that PKC-δ KO cells have decreased alcian blue staining, indicating a reduced level of glycosaminoglycan production as compared to WT mice (Fig. 5), consistent with the ex vivo observations described above. We also analyzed cell proliferation rate of primary chondrocytes in micromass cultures. The results showed that there is a decreased proliferation rate in PKC-δ KO mature chondrocytes (Fig. 5d,e). These in vitro results are consistent with the ex vivo observations presented in Fig. 2, showing diminished chondrocyte cell density.

## Discussion

The structural continuum of articular cartilage and the bone–cartilage interface is an important feature of healthy cartilage. In this study, PKC-δ KO mice were found to display structural alterations in the articular surface and the intra-articular compartment with atypical chondrocyte morphology. In the diarthrodial joint, alteration of both cartilage and subchondral bone could result in clinical joint dysfunctions in OA patients, such as joint instability, loss of motion and pain. Elucidating the role of PKC-δ in the structural regulation of the interface of bone and cartilage will give insights into our understanding of the pathogenesis of OA.

Increased subchondral bone turnover is associated with alterations at the osteochondral junction [5]. Previously, we have found that PKC-δ is an intrinsic regulator of osteoclast formation and bone resorption, and the loss of PKC-δ protects against lipopolysaccharide-induced osteolysis due to an intrinsic defect in osteoclastic bone resorption. It was also observed that the PKC-δ KO mice have areas of retained cartilage within the trabecular bone area, a characteristic of an osteopetrotic phenotype. Interestingly, in this study, we have discovered thickening of the articular cartilage surface and a significant increase in the calcein labeling intensity in the interface between the growth plate and the metaphysis. This might lead to an increased calcified compartment at the interface of bone and cartilage. These data seem to suggest a role for PKC-δ in the regulation of articular cartilage and the interface between the growth plate and metaphysis.

In line with our findings of bone and joint structural alterations in PKC-δ KO mice, previous in vitro studies have suggested that PKC-δ is a positive regulator of chondrogenesis using high-density micromass cell cultures in chicken [18]. In human adult articular chondrocytes, PKC-δ activation might be a major rate-limiting event in the basic fibroblast growth factor-dependent stimulation of matrix metalloproteinase (MMP)-13, suggesting that PKC-δ-dependent activation of multiple mitogen-activated protein kinase (MAPK) signaling pathways could contribute to OA progression [19]. Furthermore, inhibitors of PKC-δ have been shown to decrease MMP upregulation and fibronectin-mediated damage to cartilage [20].

In addition to PKC-δ, other isoforms of PKC have been implicated as having a role in chondrogenesis and OA pathogenesis. For instance, downregulation of PKC blocked both proliferation of cells and synthesis of sulfated proteoglycans, indicating that expression of both cPKCs and nPKCs is required at an early stage of chondrogenesis [21]. PKC-α and -ζ are thought to regulate NO-induced apoptosis and the dedifferentiation of articular chondrocytes [22]. PKC isoforms ζ and ι have been shown to mediate collagenase expression and cartilage destruction via STAT3- and ERK-dependent c-fos induction [23]. Activation of integrin-RACK1/PKC-α signaling is involved in human articular chondrocyte mechanotransduction [24].

Chondrocytes synthesize and maintain the extracellular matrix of articular cartilage to meet the physiological requirements of the tissue. They have a distinctive morphology and arrangement from the surface to the deep region of articular cartilage, which is important to the function of articular cartilage. Several lines of evidence have shown that the morphology of chondrocytes has a close relationship to their synthetic activities and gene expression [25, 26]. Using FOLSCM, we clearly demonstrated the morphological alterations of chondrocytes in PKC-δ KO mice. It is also worth mentioning that FOLSCM uses the same imaging technology as confocal arthroscopy that has enabled the diagnosis of microstructural differences in human patellar articular cartilage without biopsy [17]. Therefore, this study also indicates the potential of the fiber optic confocal imaging technique as a new imaging technology for direct viewing of chondrocyte morphological changes at a cellular level without biopsy.

PKC-δ is known to inhibit proliferation in B cells [7]. Surprisingly, our results showed that there is a decreased proliferation rate in PKC-δ KO mature chondrocytes. However, these in vitro results are consistent with the ex vivo observations (Fig. 2) showing a reduction in the numbers of chondrocytes in the PKC-δ KO mice. It is likely that the effect of PKC-δ on cell proliferation rate is cell type-dependent. Previous studies of PKC-δ KO mice have shown that PKC-δ is necessary for chondrocyte maturation and subsequent osteoblast differentiation during embryonic bone formation [13]. Our results further indicate an important role for PKC-δ in chondrocyte maintenance and matrix production in adulthood.

As the early cartilage degeneration progresses, OA usually affects all structures in the synovial joint. Aberrant hypertrophy and calcification have been reported in human OA with structural characteristics that resemble a terminal differentiation process during endochondral ossification [27, 28]. At a later stage, osteophytes often develop on the joint margins as a result of osseous outgrowths [29]. Subchondral bone sclerosis [30], meniscal tear and extrusion [31] and synovitis [32] may also occur due to the mechanical changes in OA cartilage, resulting in further debilitation. There has been a continuing debate as to which might come first, the changes in cartilage which causes the subchondral bone abnormality or vice versa. Interestingly, this study has shown that structural changes in articular cartilage are concomitant with the subchondral bone abnormality in PKC-δ KO mice, a phenotype similar to transforming growth factor-beta (TGF-β) KO mice [33]. In TGF-β KO mice [33], the structural alteration of articular cartilage is associated with mechanical loading. Interestingly, in PKC-δ KO mice articular cartilage, a mechanical bearing region is more affected than the growth plate, suggesting the mechanical loading might also contribute to the phenotype of PKC-δ KO mice.

## Conclusions

In this study, PKC-δ KO mice were found to display structural alterations in the articular surface, and the intra-articular compartment with atypical chondrocyte morphology, suggesting that PKC-δ plays a role in the osteochondral plasticity of the interface between the articular cartilage and osteochondral junction. Understanding the role that PKC plays in articular cartilage and the bone–cartilage interface will be important for future design of novel therapies.

## Additional file

Additional file 1:
**Safranin O staining of the growth plate in PKC-δ+/+ and PKC-δ−/− mice.** (A, B) Slightly weaker staining of safranin O was found in PKC-δ−/− mice; no significant difference in the height of the growth plate was found between PKC-δ+/+ and PKC-δ−/− mice as measured by safranin O staining regions. (C) Measurement of the height of growth plates by safranin O staining regions in PKC-δ+/+ and PKC-δ−/− mice. (TIFF 1716 kb)

## Abbreviations

DMEM: Dulbecco’s modified Eagle’s medium
FOLSCM: Fiber optic laser scanning confocal microscopy
KO: Knockout
MAPK: Mitogen-activated protein kinase
MBI: Maximum brightness image
MMA: Methylmethacrylate
MMP: Matrix metalloproteinase
NBF: Neutral buffered formalin
OA: Osteoarthritis
PBS: Phosphate-buffered saline
PKC-δ: Protein kinase C delta
ROI: Region of interest
SD: Standard deviation
TGF-β: Transforming growth factor-beta
WT: Wild-type
