# Supplementary figures and images for: Protein kinase C delta null mice exhibit structural alterations in articular surface, intra-articular and subchondral compartments
Source: Arthritis Res Ther. 2015 Aug 17;17(1):210. doi: 10.1186/s13075-015-0720-4 (PMC4538913; doi:10.1186/s13075-015-0720-4)

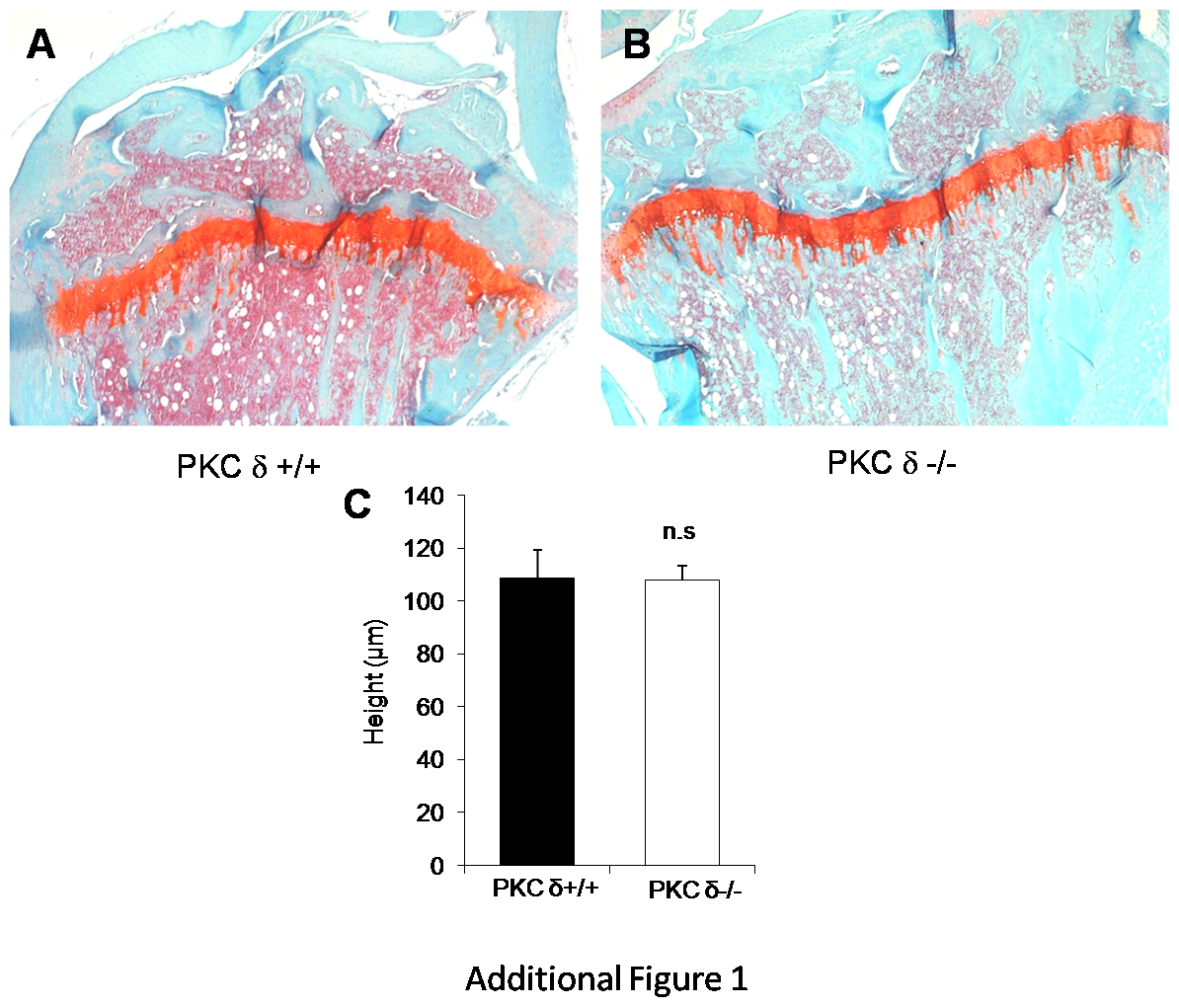

Supplement: Additional file 1: — Safranin O staining of the growth plate in PKC-δ+/+ and PKC-δ−/− mice. (A, B) Slightly weaker staining of safranin O was found in PKC-δ−/− mice; no significant difference in the height of the growth plate was found between PKC-δ+/+ and PKC-δ−/− mice as measured by safranin O staining regions. (C) Measurement of the height of growth plates by safranin O staining regions in PKC-δ+/+ and PKC-δ−/− mice. (TIFF 1716 kb) [file 13075_2015_720_MOESM1_ESM.tiff]
